# Single cell transcriptomic analyses reveal diverse and dynamic changes of distinct populations of lung interstitial macrophages in hypoxia-induced pulmonary hypertension

**DOI:** 10.3389/fimmu.2024.1372959

**Published:** 2024-04-15

**Authors:** Sushil Kumar, Claudia Mickael, Rahul Kumar, Ram Raj Prasad, Nzali V. Campbell, Hui Zhang, Min Li, B. Alexandre McKeon, Thaddeus E. Allen, Brian B. Graham, Yen-Rei A. Yu, Kurt R. Stenmark

**Affiliations:** ^1^ Department of Pediatrics and Cardiovascular Pulmonary Research Laboratory, University of Colorado School of Medicine, Aurora, CO, United States; ^2^ Division of Pulmonary Sciences and Critical Care Medicine, Cardiovascular Pulmonary Research Laboratory, University of Colorado School of Medicine, Aurora, CO, United States; ^3^ Department of Medicine, University of California San Francisco, San Francisco, CA, United States; ^4^ Lung Biology Center, Zuckerberg San Francisco General Hospital, San Francisco, CA, United States

**Keywords:** interstitial macrophages, vascular remodeling, pulmonary hypertension, hypoxia, complement, inflammation

## Abstract

**Introduction:**

Hypoxia is a common pathological driver contributing to various forms of pulmonary vascular diseases leading to pulmonary hypertension (PH). Pulmonary interstitial macrophages (IMs) play pivotal roles in immune and vascular dysfunction, leading to inflammation, abnormal remodeling, and fibrosis in PH. However, IMs’ response to hypoxia and their role in PH progression remain largely unknown. We utilized a murine model of hypoxia-induced PH to investigate the repertoire and functional profiles of IMs in response to acute and prolonged hypoxia, aiming to elucidate their contributions to PH development.

**Methods:**

We conducted single-cell transcriptomic analyses to characterize the repertoire and functional profiles of murine pulmonary IMs following exposure to hypobaric hypoxia for varying durations (0, 1, 3, 7, and 21 days). Hallmark pathways from the mouse Molecular Signatures Database were utilized to characterize the molecular function of the IM subpopulation in response to hypoxia.

**Results:**

Our analysis revealed an early acute inflammatory phase during acute hypoxia exposure (Days 1-3), which was resolved by Day 7, followed by a pro-remodeling phase during prolonged hypoxia (Days 7-21). These phases were marked by distinct subpopulations of IMs: MHCII^hi^CCR2^+^EAR2^+^ cells characterized the acute inflammatory phase, while TLF^+^VCAM1^hi^ cells dominated the pro-remodeling phase. The acute inflammatory phase exhibited enrichment in interferon-gamma, IL-2, and IL-6 pathways, while the pro-remodeling phase showed dysregulated chemokine production, hemoglobin clearance, and tissue repair profiles, along with activation of distinct complement pathways.

**Discussion:**

Our findings demonstrate the existence of distinct populations of pulmonary interstitial macrophages corresponding to acute and prolonged hypoxia exposure, pivotal in regulating the inflammatory and remodeling phases of PH pathogenesis. This understanding offers potential avenues for targeted interventions, tailored to specific populations and distinct phases of the disease. Moreover, further identification of triggers for pro-remodeling IMs holds promise in unveiling novel therapeutic strategies for pulmonary hypertension.

## Introduction

Pulmonary hypertension (PH) is a heterogeneous group of diseases characterized by elevated pulmonary arterial pressures as a result of pulmonary vascular inflammation and remodeling, eventually leading to right heart failure and death (1–4). In PH, hypoxia and immune dysfunction are central contributors to disease pathogenesis (5–10). The mechanisms through which hypoxia and immune dysfunction contribute to PH have been examined in animal models, including hypoxia-induced PH (11, 12). Myeloid leukocytes, especially macrophages, are thought to be the primary effectors of inflammation and remodeling in the PH vessel wall (1, 5). However, the transcriptional repertoire and functional profiles of pulmonary interstitial macrophage (IM) in response to hypoxia that regulate abnormal pulmonary vascular remodeling remain largely unknown.

Under normal homeostatic conditions, pulmonary IMs comprise a heterogeneous population of cells that can arise from embryonic origins with local self-renewing capacities or are recruited from circulating monocytes (13–15). Depending on the stimuli and microenvironment, IMs’ repertoire and functional profiles in disease are altered. Systemic and tissue hypoxia can shape the immune landscape and macrophage function to regulate lung injury and repair. In animal models of PH, prolonged hypoxia causes a marked accumulation of monocytes and macrophages, especially in the perivascular regions of the lung’s blood vessels, leading to vascular thickening and elevation of pulmonary pressures (5, 16–19). Prior studies using bulk transcriptomic analyses of pulmonary macrophages in a hypoxia-induced PH model showed dynamic time- and compartment-specific changes in IMs in response to hypoxia exposure (17). Studies have also demonstrated that pulmonary IMs are central to the development of PH (17, 20). Thus, hypoxia contributes to PH, at least in part, via its effects on pulmonary IMs. However, an understanding of when and how hypoxia affects pulmonary IM population gene expression and function has previously been hindered by significant marker overlaps among the subsets. The advent of single-cell RNA sequencing (scRNAseq) allows for identifying and characterizing pulmonary macrophages under homeostatic and in response to injuries and environmental stimuli (13, 15).

This study aims to elucidate the effects of hypoxia on pulmonary IM repertoire and their functional states using a murine model of hypoxic PH. We employed scRNAseq to examine dynamic temporal changes in IM populations and gene expression patterns. Understanding how the transcriptional profiles of interstitial macrophages evolve in response to hypoxia or disease progression is crucial for decoding their roles in maintaining tissue function or contributing to PH. This information is invaluable for elucidating the contributions of interstitial macrophages to disease pathogenesis. It can potentially guide the development of targeted therapeutic strategies to modulate the functions of distinct macrophage subpopulations.

## Materials and methods

### Animal and cell preparation

Animal procedures adhered to protocols approved by the University of Colorado Institutional Animal Care and Use Committee. Cx3cr1^+/GFP^ reporter mice [B6.129P2(Cg)-Cx3cr1tm1Litt/J, Jackson Labs Stock No.: 005582] were bred with C57BL/6J (Jackson Stock No. 000664) and were raised at an altitude of 1,609 meters (Denver altitude). We have previously noted mild changes in pulmonary interstitial macrophage transcriptomic profiles in animals at Denver altitude compared to sea level (**Supplementary Figures S1A–F**). To avoid potential confounders of adaptation due to residence in reduced oxygen at altitude, Cx3cr1^+/GFP^ animals were placed in sea level (~21% fraction of inspired oxygen (FiO2)) chamber at 5 weeks of age for 4 weeks to allow acclimation to sea level condition. Subsequently, animals were maintained at sea level (Day 0) or exposed to hypoxia conditions (simulated altitude of 5,486 meters, in the hypobaric chamber; ~10% FiO2) for 1, 3, 7, or 21 days.

For each time point, the lungs of a pair of age and sex-matched mice (1 male and 1 female) were harvested for subsequent analyses as previously described (21). Briefly, animals were anesthetized using a ketamine and xylazine mixture at final concentrations of 100 mg/kg and 20 mg/kg, respectively. To distinguish between interstitial and circulating cells, mice were administered a retro-orbital injection of an anti-CD45 antibody (1 µg/mouse) 5 minutes before euthanasia to label intravascular leukocytes. Afterward, the lungs were perfused via the right ventricle with PBS. The PBS-flushed lungs were then collected and individually subjected to digestion. Briefly, perfused lung tissues underwent enzymatic digestion using liberase (Roche, Germany) dissolved in RPMI medium (Mediatech, Corning, NY) at a concentration of 1 mg/ml, for 30 minutes at 37°C. The tissue underwent further mechanical disruption by passing through 16-gauge and 18-gauge needles five times each. Afterward, the cells were filtered through a 100 μm cell strainer (Fisher Scientific) and centrifuged for 5 minutes at 400x g. Red blood cells (RBCs) were lysed using 1 ml of ACK lysis buffer (Gibco). Following RBC lysis, the cells were resuspended and washed in RPMI to neutralize the lysis buffer. As previously described, pulmonary interstitial macrophages were sorted by flow cytometry (22). Briefly, singlet cells were gated as CD45^+^ and Lin^-^ (excluding T cells, B cells, natural killer cells, and neutrophils). Intravascular cells were excluded by negative gating for intravenous anti-CD45. Subsequently, cells were gated based on GFP^hi^ expression, followed by CD64^+^ and CD11b^+^ expression for IMs, while AMs were gated as GFP^lo^ CD64^+^ CD11c^+^. Flow sorting was conducted using an Astrios EQ cell sorter (Beckman Coulter Life Sciences) at the CUAMC Cancer Center Flow Cytometry Shared Resource. Post-sorting, isolated cells were collected in a specialized medium (HBSS-Gibco with 2.5% FBS), centrifuged, accurately counted, and prepared for subsequent sequencing analysis. In each experimental group, IMs were sorted from one male and one female mouse, pooled, and utilized for library preparation and sequencing using the 10x Genomics platform according to standard protocols described below.Single-cell RNA sequencing

#### Cell capture and library preparation

For each time point, sorted pulmonary interstitial macrophages from at least two animals (male and female) were combined for single-cell RNA sequencing. The CUAMC Genomics Core conducted the sequencing process, wherein the suspension of sorted IMs was loaded onto the Chromium Single Cell Controller (10x Genomics) using the Single-Cell 3′ Library and Gel Bead Kit V3.1 (10x Genomics, 1000268) alongside the Chromium Single Cell G Chip Kit (10x Genomics, 1000120) to generate single-cell gel beads in the emulsion as per the manufacturer’s protocol. In brief, single cells were suspended in phosphate-buffered saline containing 0.04% bovine serum albumin. Approximately 10,000 cells were introduced into each channel, aiming to recover an estimated 5,000 cells. Captured cells were then lysed, releasing RNA, which was barcoded through reverse transcription within individual Gel Bead-In Emulsions (GEMs). Reverse transcription was carried out on an S1000TM Touch Thermal Cycler (Bio-Rad) at 53°C for 45 minutes, followed by 85°C for 5 minutes, and then held at 4°C. The resulting cDNA was amplified, and its quality was assessed using an Agilent 4200 system. ScRNAseq libraries were constructed following the manufacturer’s instructions using the Single Cell 3′ Library and Gel Bead Kit V3.1. Finally, the libraries were sequenced on an Illumina NovaSeq 6000 sequencer with a sequencing depth of at least 100,000 reads per cell using the paired-end 150 bp strategy.

#### Sequence alignment and quality control

We aligned sequence reads to the *Mus musculus* GRCm39 reference genome using Cell Ranger v6 (10x Genomics). The raw read counts from all time points were aggregated and subjected to downstream analysis using Seurat (v4.3.0) (23). Low-quality cells, doublets, and damaged cells defined as having unique feature count < 300 (low quality) or >5,000 (doublets), and >20% mitochondria content (damaged/stressed cells) were excluded from the dataset using Seurat and DoubletFinder (v2.0.3) (24) The dot plot and bar plot visualizations were produced using ggplot2 (v3.4.3), while the heatmap visualization for average gene expression and pathways was created using the ComplexHeatmap (v2.14.0) library in R (v4.2.2).

#### Read counts normalization and scaling

Filtered gene expression measurements were normalized using the LogNormalize method with scale.factor = 10000, followed by selecting the top 2000 highly variable genes with ‘vst’ selection method. Thereafter, data was scaled using the ScaleData method with regression of unwanted sources of variation such as mitochondria content, feature count, and cell cycle phage. The scaled data was used to analyze the initial 100 dimensions of PCA. Subsequently, the significant dimensions (1 to 18) were utilized for neighbor identification and cluster analysis, employing Uniform Manifold Approximation and Projection (UMAP) with the ‘umap-learn’ method (23).

#### Data integration

Data integration was performed using the ‘IntegrateData’ function and the reciprocal principal component analysis (rPCA) method with ‘k.anchor=5’ of the Seurat library to integrate data of all timepoint and remove batch effects. The integrated data underwent reclustering with the indicated parameter (20).

#### Cell Identification and cluster assignment

We employed the SingleR (v1.6.1) (https://github.com/dviraran/SingleR) to annotate cell types in our scRNAseq data by comparing individual cell gene expression profiles with ImmGen (Immunological Genome Project) datasets for cell type assignment (**Supplementary Figures S2A, B**). Of the identified cells, less than 6% were non-macrophage cells, including monocytes, dendritic cells, T-cells, B-cells, NKT cells, and endothelial cells. Non-macrophages were subsequently excluded from downstream analysis. Clustering using shared nearest neighbors (SNN) with the k-means 2 and 18 was applied to identify the IM subpopulations. Temporal clusters were merged based on a similar cell proportion pattern.

#### Differential gene expression and pathways analyses

For cluster-specific gene identification, the ‘FindAllMarkers’ function, while for conserved cluster-specific genes identification, the ‘FindConservedMarkers’ function was employed. To identify differentially expressed genes (DEGs) for each time point compared to day 0 within each cluster, the ‘FindMarkers’ function was used, followed by the identification of positive and negative (differentially expressed gene) DEGs using a nonparametric Wilcoxon rank-sum test with a significance threshold (q value) < 0.05. GSVA (v1.46.0) (25) and the FGSEA (v1.18.0) (26) library in R were employed to perform gene set variation analysis (GSVA) and gene set enrichment analysis (GSEA). Hallmark pathways from the mouse Molecular Signatures Database (MSigDB) were utilized for GSVA analysis of the IM subpopulation and GSEA analysis to understand the response at different time points of hypoxia. Adjusted p-value < 0.05 and positive normalized enrichment score (NES) are considered enriched pathways.

### Immunofluorescence

Murine lung tissues were perfused with phosphate-buffered-saline (PBS) solution, inflated with 4% paraformaldehyde (PFA) (Sigma Aldrich, St. Louise, MO), and fixed for 2 hours. Fixed tissues were washed with PBS solution and treated with 30% sucrose solution. Treated tissues were embedded in Optimal Cutting Temperature compound (OCT). Frozen tissue sections of 6-8µm were prepared. Immunofluorescence staining was performed using rat anti-mouse CD64 (AT152-9; Bio-Rad, Hercules, CA), mouse anti-α-smooth muscle actin-FITC (SMA-FITC)(1A4; Sigma Aldrich), and rabbit anti-mouse VCAM1 (SA05-04; Thermo Fisher Scientific). 4’,6’-diamidino-2-phenylindole (DAPI) was used for nuclear staining. CD64, VCAM1, and SMA-FITC staining were performed using tyramide amplification (Akoya Bioscience TSA Plus kits). Small pulmonary arteries of similar sizes were selected blindly across treatment or animal groups for imaging. Confocal images were obtained with a Olympus FV1000 laser scanning confocal microscope using 10x objective (Zeiss, Cambridge, UK). The confocal images underwent processing and quantification of VCAM1^hi^ macrophage using FIJI ImageJ (National Institute of Health).

## Results

### Animals acclimated to sea level share similar IM subpopulations and transcriptional profiles as those raised at sea level locations

**Figure 1:**
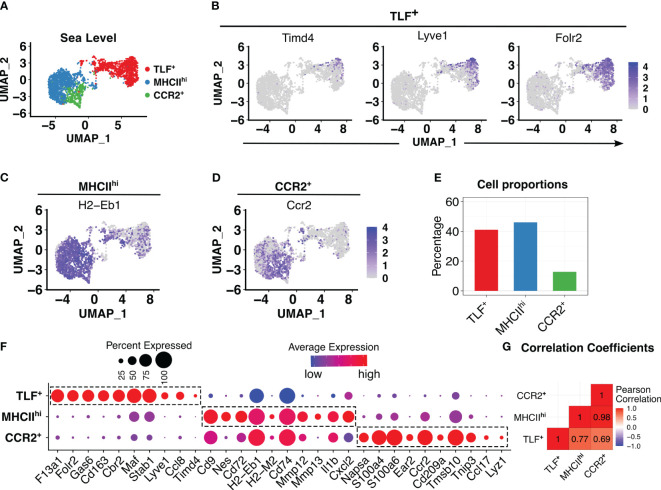
In animals acclimated to sea level, three distinct interstitial macrophage (IM) subpopulations were observed. **(A)** PCA plot showed three distinct IM subpopulations. **(B-D)** Feature plots showing expression of cluster-defining markers: TLF^+^ (*Timd4*, *Lyve1*, and *Folr2*), MHCII^hi^ (*H2-Eb1*), and CCR2^+^ (*Ccr2*). **(E)** Bar plots depicting percentages of each subpopulation as a proportion of IMs. **(F)** Dot plot showing average and percentage expression of top 10 cluster defining genes for each of three subpopulations. **(G)** Pearson correlation analysis highlights a transcriptional difference in the TLF^+^ subpopulation from MHCII^hi^ and CCR2^+^, but shared gene profiles between MHCII^hi^ and CCR2^+^ subpopulations.

After conducting quality control and excluding non-macrophages, 2521 pulmonary interstitial macrophages were identified in the sea level (Day 0) sample. Similar to previously described, clustering analyses revealed three transcriptionally distinct populations: TLF^+^ (Timd4, Lyve1, and Folr2), MHCII^hi^, and CCR2^+^ (**Figure 1A**) (15). The TLF cluster expresses Timd4, Lyve1, and Folr2 genes (**Figure 1B**, **Supplementary Figures S3A, B**) (15). *Ccr2* expression was highest in the CCR2^+^ cluster. *H2-Eb1*, which encodes for histocompatibility class II antigen (MHCII), was expressed in the MHCII^hi^ and CCR2^+^ clusters (**Figures 1C, D**). *Ccr2* expression was highest in the CCR2^+^ cluster. *H2-Eb1*, which encodes for histocompatibility class II antigen (MHCII), was expressed in the MHCII^hi^ and CCR2^+^ clusters (**Figures 1C, D**). MHCII^hi^ and TLF^+^ clusters comprised the majority of IM examined (46.1% and 41.1%, respectively), and the CCR2^+^ cluster represented the smallest cluster (12.8%) (**Figure 1E**). To exclude sex as a potential confounder in the data analyses, we distinguished IMs derived from male *vs.* female mice using Y-chromosome-associated gene transcripts and X-inactive specific transcripts (Xist) RNA. There were no significant differences in IM distribution between the sexes within this dataset that may confound the findings (**Supplementary Figure S3C**).

While cluster-defining marker analyses revealed three transcriptionally distinct populations as previously described, there were significant overlapping gene expression patterns between the MHCII^hi^ and CCR2^+^ subpopulations (**Figure 1F**, **Supplementary Figures S3A, B**) suggesting MHCII^hi^ and CCR2^+^ subpopulations are highly related. Pearson method for correlation coefficient analysis of IM subpopulations confirms this observation: while the TLF^+^ subpopulation exhibited a significant transcriptional distinction from both the MHCII^hi^ (correlation coefficient 0.77) and CCR2^+^ (correlation coefficient 0.69) subpopulations, there was a high correlation coefficient of 0.98 between MHCII^hi^ and CCR2^+^ (**Figure 1G**). Thus, MHCII^hi^ and CCR2^+^ populations are highly related.

### Dynamic changes in IM populations are observed with hypoxia exposure

**Figure 2:**
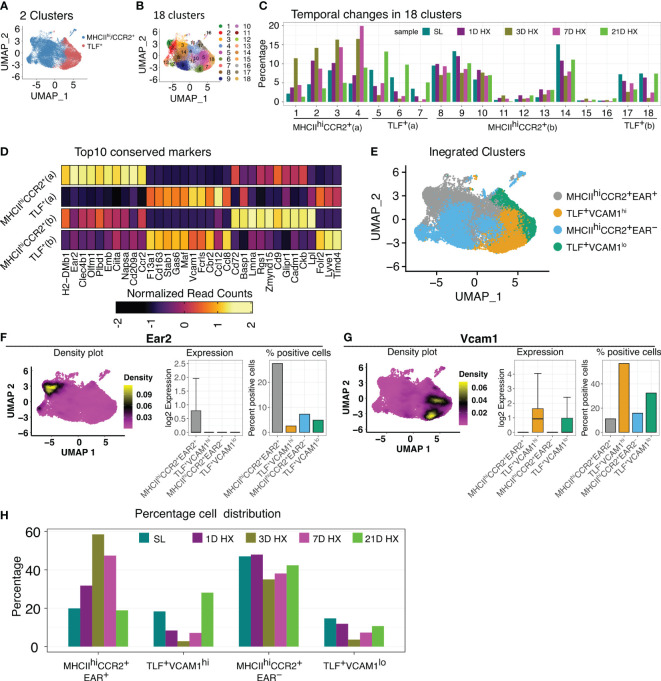
Dynamic IM population changes in response to hypoxia. (**A, B**) UMAP visualization of integrated data (SL, Days 1, 3, 7, and 21 of hypoxia exposure) representing 2 clusters and 18 clusters. **(C)** Bar graph depicting the 4 patterns of cell proportion changes throughout the course of hypoxia exposure among 18 subclusters: MHCII^hi^CCR2^+^(a), MHCII^hi^CCR2^+^(b), TLF^+^(a), and TLF^+^(b). **(D)** Heatmap showing the expression of the top 10 conserved markers for each temporal subcluster. **(E)** UMAP visualization of the four distinct temporal cell populations. **(F)** The density plot illustrates that *Ear2* expression is predominantly localized within the MHCII^hi^CCR2^+^EAR2^+^ cluster. Bar graphs depicting the level and percent of *Ear2* expression level in each subcluster. **(G)** The density plot illustrates that *Vcam1* expression is predominantly localized within the TLF^+^VCAM1^hi^ cluster. Bar graphs depicting the level and percent of *Vcam1* expression level in each subcluster. **(H)** Bar graph showing the distribution of cell percentage of four clusters over the course of hypoxia exposure.

To evaluate potential hypoxia-induced responses, we integrated cells from various time points, encompassing sea level (day 0) and days 1, 3, 7, and 21 of hypoxic exposure. Initial sub-clustering analyses identified two large clusters reflecting TLF^+^ and MHCII^hi^CCR2^+^ populations (**Figure 2A**) but did not capture the dynamic accumulation of a distinct population of inflammatory macrophages in response hypoxia exposure previously described by flow cytometry (8, 27). A sub-clustering approach was applied to examine potential dynamic cellular changes, identifying 18 subclusters (**Figure 2B**). Four patterns of temporal cellular dynamics in the subclusters were identified (**Figure 2C**). Group 1 (subclusters 1, 2, 3, and 4; MHCII^hi^CCR2^+^ (a)) derived from MHCII^hi^CCR2^+^ cluster showing an increase in cell proportions at day 1, reaching their highest levels at day 3, followed by a decrease in cell proportion at day 7, and ultimately approaching the cell proportions observed at day 21 to sea level (**Figure 2C**). Group 2 (subclusters 5, 6, and 7; TLF^+^ (a)) derived from the TLF+ cluster showed a decrease in cell proportions at day 1 of hypoxia exposure, reaching their lowest levels at day 3 hypoxia, followed by an increase in cell numbers at day 7 hypoxia, and ultimately accumulated at significantly higher cell proportions compared to sea level by day 21 (**Figure 2C**). Group 3 (subclusters 8-16 derived from MHCII^hi^CCR2^+^ cluster; MHCII^hi^CCR2^+^ (b)) and Group 4 (subclusters 17 and 18 arise from TLF^+^ cluster; TLF^+^ (b)) remained largely consistent in their cellular dynamics in response to hypoxia except cluster 18 showed decreased cell proportion at day 3 and day 7 (**Figure 2C**). Cluster stability analyses showed a general increase in the stability of aggregated clusters compared to individual subclusters, confirming the reliability of the aggregated groupings (**Supplementary Figure S4**). Thus, the aggregation of subclusters resulted in four distinct stable clusters reflecting temporal changes in IM populations in response to hypoxia exposure: MHCII^hi^CCR2^+^ (a), TLF^+^ (a), MHCII^hi^CCR2^+^ (b), and TLF^+^ (b) (**Figure 2C**).

### Four molecularly distinct IM clusters were defined in response to hypoxia exposure

Conserved marker analyses revealed that MHCII^hi^CCR2^+^ (a) uniquely expresses Ear2, and DEG analysis showed an exclusively high level of *Ear2* expression in the MHCII^hi^CCR2^+^ (a) population (**Figures 2D–F**). Consequently, we labeled MHCII^hi^CCR2^+^ (a) as MHCII^hi^CCR2^+^EAR2^+^ and MHCII^hi^CCR2^+^ (b) as MHCII^hi^CCR2^+^EAR2^-^ (**Figures 2D–F**). Similarly, we noticed that TLF^+^ (a) exhibited increased *Vcam1* positivity, with the other clusters showing low *Vcam1* expression (**Figures 2D, E, G**). We designated TLF^+^ (a) as TLF^+^VCAM1^hi^ and TLF^+^ (b) as TLF^+^VCAM1^lo^ (**Figures 2D, E, G**). The characteristics of the MHCII^hi^CCR2^+^EAR2^-^ and TLF^+^VCAM1^lo^ clusters are shown by dot plots and density plots (**Supplementary Figures 5SA–D**). The sub-clustering approach captured dynamic IM changes and resulted in four molecularly distinct populations of IMs (**Figure 2H**).

These newly defined clusters detailed the dynamic IM population changes in response to hypoxia exposure. Proportions of cells in MHCII^hi^CCR2^+^EAR2^-^ and TLF^+^VCAM1^lo^ decreased on day 3 of hypoxia, but subsequently, both subpopulations began to increase on days 7; and by day 21, they approach that of sea level proportions (**Figure 2H**). The proportion of MHCII^hi^CCR2^+^EAR2^+^ cells stood at 19.9% at sea level. Following one day of acute hypoxic exposure, this percentage increased to 31.8%. It reached its zenith at 58.5% after three days of acute hypoxia exposure. Subsequently, it declined to 47.4% after seven days of hypoxia exposure and eventually settled at 18.9% after 21 days, which was close to the initial sea level percentage of 19.9% (**Figure 2H**).

In contrast, the proportion of the TLF^+^VCAM1^hi^ subpopulation decreased in response to acute hypoxia exposure (from ~18% at level to 10% on day 1). The cell proportions continued to decline, reaching a minimum of 5% on day 3 of hypoxia. Then, the cell proportion begins to increase on day 7. By day 21 of hypoxia exposure, the TLF^+^VCAM1^hi^ proportion exceeded that of sea level (from ~18% at sea level to ~28% on Day 21) (**Figure 2H**). These findings demonstrate that dynamic and distinct IM populations emerge in response to acute *vs.* prolonged hypoxia.

**Figure 3:**
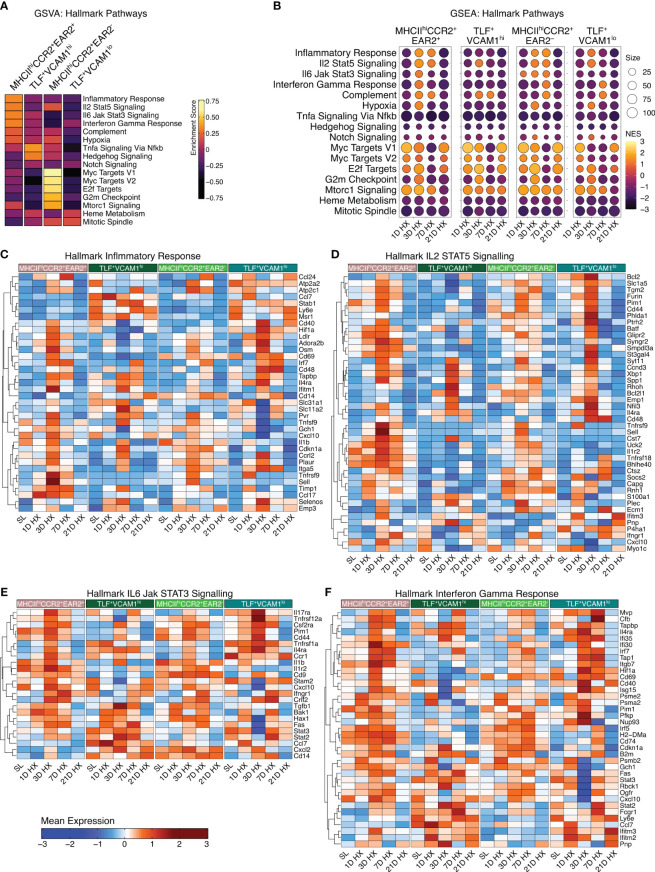
Enrichment of hallmark inflammatory response-associated pathways. **(A)** Gene Set Variation Analysis (GSVA) demonstrates cluster-specific hallmark pathway activation. **(B)** The dot plots visually depict Gene Set Enrichment Analysis (GSEA), demonstrating pathway enrichment at each time point compared to sea level. Dot size corresponds to the number of genes matched with Hallmark pathways. The dot color indicates the normalized enrichment score (NES). **(C–F)** Heatmaps depict gene expression profiles for significantly enriched inflammation-associated pathways, including Hallmark Inflammatory Response, Hallmark IL2 STAT5 Signaling, Hallmark IL6 Jak STAT3 Signaling, and Hallmark Interferon Gamma Response.

With dynamic temporal and cellular changes in four groups of IMs suggesting distinct roles in regulating vascular remodeling processes and the development of PH, we conducted gene set variation analysis (GSVA) to identify Hallmark pathways for each subpopulation based on absolute expression levels. MHCII^hi^CCR2^+^EAR2^-^ enriched in Myc Targets V1 and V2, E2F Targets, G2M Checkpoint, and mTORC1 Signaling, and TLF^+^VCAM1^lo^ population exhibited enrichment in Heme Metabolism and Mitotic Spindle pathways (**Figure 3A**) suggestive for these two populations having a role in regulating homeostatic functions. Corresponding to their significant increase in cell number in response to acute hypoxia exposure (Day 1-3), the MHCII^hi^CCR2^+^EAR2^+^ population was highly enriched for pathways related to acute hypoxia and type 1 acute inflammatory activation (*i.e.*, Inflammatory Response, IL2 STAT5 Signaling, IL6 Jak Signaling, Interferon Gamma Response, Complement, and hypoxia) (**Figure 3A**). On the other hand, the TLF^+^VCAM1^hi^ population showed enrichment in TNFα Signaling via NFκB, Hedgehog Signaling, and Notch Signaling, which are known pathways associated with vascular development and remodeling (28–30). These findings reflect waves of IMs with distinct functional roles contributing to the development of hypoxia-induced PH.

### Acute hypoxia exposure induces transient acute inflammatory responses in IMs

MHCII^hi^CCR2^+^EAR2^+^ exhibited the highest enrichment in cellular proportion and upregulation of pathways associated with inflammatory responses to acute hypoxia exposure (Day 1-3), but these inflammatory responses began to wean by day 7. By day 21, inflammatory signals and gene expressions largely resemble that of sea level (**Figure 3B**). While the inflammatory signals were most pronounced in the MHCII^hi^CCR2^+^EAR2^+^, MHCII^hi^CCR2^+^EAR2^-^ and TLF^+^VCAM1^lo^ populations similarly upregulate Type 1 inflammatory associated pathways between day 1 and 3 (i.e., Inflammatory response, IL2 STAT5 signaling, IL6 Jak Stat3 signaling, Interferon Gamma responses, Complement, and Hypoxia) (**Figure 3B**). These pathways associated with acute hypoxia and inflammation were down-regulated (day 7) and returned to sea level by day 21 (**Figures 3B–F**). These findings are consistent with IM populations mounting acute Type 1 inflammatory response to acute hypoxic stress followed by resolution of acute inflammation despite continued hypoxic exposure.

### Emergence of dysregulated TLF^+^VCAM1^hi^ IMs with prolonged hypoxia exposure

**Figure 4:**
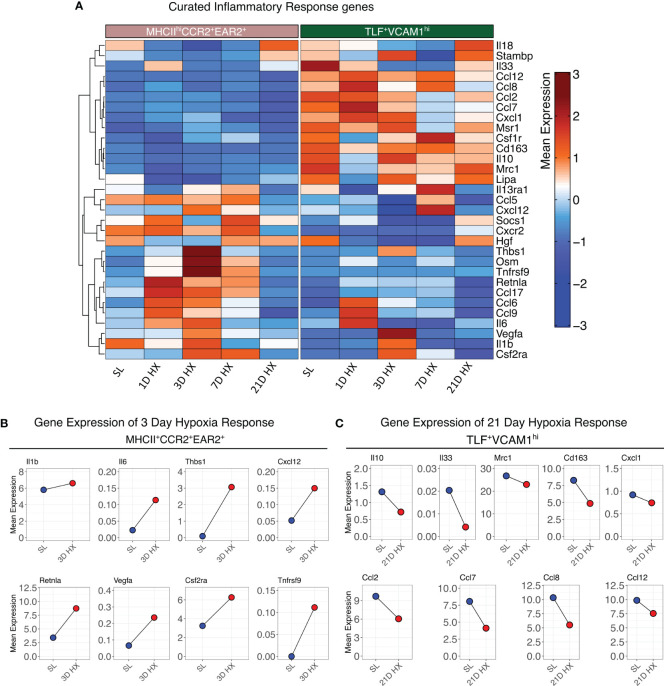
Distinct functional profiles between MHCII^hi^CCR2^+^EAR2^+^ and TLF^+^VCAM1^hi^ populations. **(A)** Heatmaps depicting the gene expression profiles of curated inflammatory response genes. **(B)** Dot plots illustrate the absolute mean expression of key genes in the SL and 3D hypoxia of the MHCII^hi^CCR2^+^EAR2^+^ subpopulation. **(C)** Dot plot illustrating the absolute mean expression of key genes in the SL and 21D hypoxia of TLF^+^VCAM1^hi^ subpopulation.

**Figure 5:**
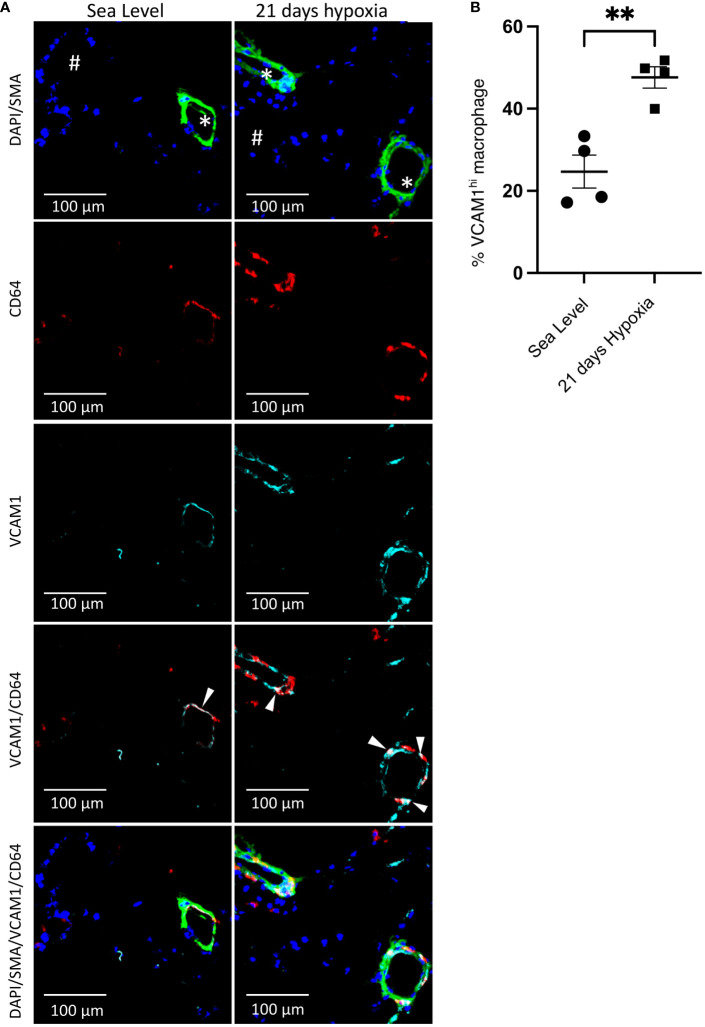
Accumulation of perivascular VCAM1-expressing macrophages after 21 days of hypoxia exposure. **(A)** Representative immunofluorescence illustration of mouse lung tissue. First row: The merged immunostaining image of DAPI (blue) and SMA (green) illustrates the artery. Second and third rows: Illustrating CD64 (red) and VCAM1 (cyan), emphasizing the presence of perivascular CD64^+^VCAM1-expressing macrophages in the lungs of wild-type mice at sea level and following 21 days of hypoxia exposure. Fourth row: The merged image for CD64 (red) and VCAM1 (cyan) demonstrates overlapping regions, as indicated by the white arrowheads, confirming the presence of VCAM1-expressing macrophages in the artery. Fifth row: Demonstrates the merge of all images together. **(B)** Quantitative assessment of VCAM1-expressing macrophages within the perivascular region of the artery. Unpaired t test with Welch’s correction was used for comparison between Sea Level and 21 days hypoxia. Scale bars indicate 100 μm. # = airway; * = pulmonary artery; SMA, Smooth Muscle Actin; **p-value < 0.01. Images are representative of n=4/group of animals analyzed.

Contrasting the acute inflammatory and hypoxia responses observed in MHCII^hi^CCR2^+^EAR2^+^, MHCII^hi^CCR2^+^EAR2^-^, and TLF^+^VCAM1^lo^ populations, the TLF^+^VCAM1^hi^ population exhibited more limited acute type 1 inflammatory and hypoxia reactions to acute hypoxia exposure (**Figures 3B-F**, **4A, B**). As described above, TLF^+^VCAM1^hi^ IMs were enriched for pathways associated with regulating vascular development and remodeling, suggesting that they have a significant role in regulating the vascular microenvironment (**Figure 3A**). At sea level, TLF^+^VCAM1^hi^ IMs express high levels of *Ccl2*, *Ccl7*, *Ccl8*, *Ccl12, Cxcl1* which are ligands for CCR1, CCR2, CCR5, and CXCL2 chemokine receptors that can regulate vast arrays of parenchymal and immune cell trafficking, migration, and functions central to immunosurveillance and vascular health (**Figure 4A**). They also express high levels of CD163, a receptor central to macrophage-mediate clearance of hemoglobin/haptoglobin complexes that would otherwise cause vasoconstriction and vascular injury (**Figure 4A**) (31). Together with high levels of *Cd206/Mrc1*, *Il10*, and *Il33* expression, the TLF^+^VCAM1^hi^ transcriptomic profile is consistent with their central immunomodulatory role in balancing vascular inflammation *vs.* repair to maintain homeostasis (**Figure 4A**) (32). However, by day 21 of prolonged hypoxia exposure, *Ccl2*, *Ccl7*, *Ccl8*, *Ccl12, and Cxcl1* chemokine expressions in TLF^+^VCAM1^hi^ IMs were down-regulated (**Figure 4C**). Similarly, compared to sea level, *Cd163*, *Cd206/Mrc1*, *Il33*, and *Il10* expressions also decreased at day 21 (**Figure 4C**). To examine whether VCAM1-expressing macrophages are detected around pulmonary arterioles, we performed immunofluorescent staining of lung tissues derived from sea level and 21 days of hypoxia exposure (**Figure 5**). We observed increased accumulation of macrophages (CD64^+^) expressing VCAM1 accumulated around remodeled pulmonary arterioles, further suggesting a role for TLF^+^VCAM1^hi^ macrophages in regulating pulmonary vascular remodeling (**Figure 5**). Together, these findings are consistent with the emergence of a dysregulated TLF^+^VCAM1^hi^ population coordinating the development of pulmonary vascular remodeling.

### Classical complement pathway upregulation in TLF^+^VCAM1^hi^ IMs after chronic hypoxia exposure

**Figure 6:**
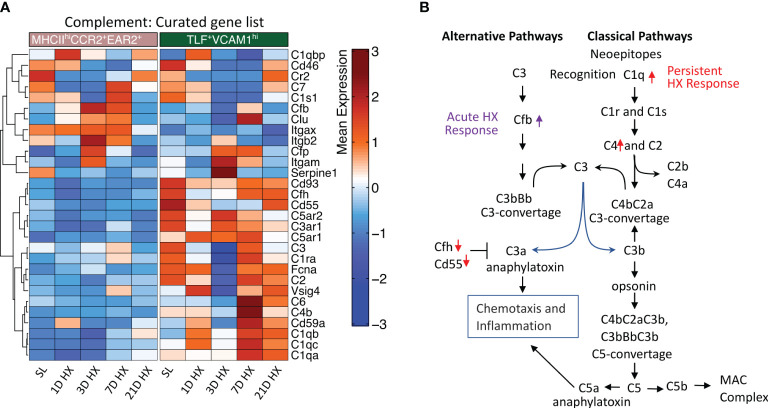
Distinct activation of complement pathways between MHCII^hi^CCR2^+^EAR2^+^ and TLF^+^VCAM1^hi^ populations. **(A)** Heatmaps show differentially expressed genes associated with complement pathways in the MHCIIhiCCR2+EAR2+ and TLF+VCAM1hi populations. **(B)** Schema showing genes associated with key regulators of complement pathways in MHCII^hi^CCR2^+^EAR2^+^ and TLF^+^VCAM1^hi^ population in response to acute and prolonged hypoxia exposure.

We have previously described complement deposition and activation as associated with the development of hypoxia-induced PH (33). Specifically, activation of the complement alternative pathway is a key mechanism initiating pro-inflammatory processes in the early stage of experimental hypoxic PH (33). Consistent with this observation, the MHCII^hi^CCR2^+^EAR2^+^ population arising during days 1-3 of acute hypoxia exposure exhibited the strongest acute pro-inflammatory signals, also upregulated *Cfb* expression, a key activator of the alternative complement pathway (**Figures 6A, B**, **Supplementary Figure S6**). These findings suggest MHCII^hi^CCR2^+^EAR2^+^ IMs are a cell population that can regulate complement alternative pathway activation and augment pro-inflammatory processes in the early stage of hypoxia-induced PH.

Contrasting MHCII^hi^CCR2^+^EAR2^+^ cells, expression of genes associated with classical complement pathway activation (i.e., *C1qa, C1qb, C1qc, and C4*) were upregulated and inhibitors (i.e., *Cfh* and *Cd55*) were down-regulated in dysregulated TLF^+^VCAM1^hi^ population at Day 7 and 21 linking classical complement pathway activations to vascular remodeling cell profile and prolonged hypoxic microenvironment (**Figures 6A, B**).

## Discussion

Hypoxia is one of the key driving factors in pulmonary vascular disease leading to pulmonary hypertension through its’ immunomodulatory effects on immune cells, including pulmonary macrophages. We and others have demonstrated a central role of pulmonary IMs in the development and progression of PH (8, 17, 20). However, how hypoxia shapes pulmonary IM transcriptional and functional profiles leading to PH development has not been extensively examined. Using a murine model of hypoxia-induced PH and single-cell transcriptomic analyses, we defined the pulmonary IM transcriptional and functional profiles over a period of 21 days of hypoxia exposure. We observed type 1 acute inflammatory activation in most subpopulations of IMs (*i.e.*, MHCII^hi^CCR2^+^EAR2^+^, MCHII^+^CCR2^+^EAR2^-^, TLF^+^VCAM1^lo^) that peaked around Day 3 and resolved almost entirely by Day 7 of acute hypoxia exposure. Large numbers of MHCII^hi^CCR2^+^EAR2^+^ cells, demonstrating the highest pro-inflammatory potential and activation of the alternative complement pathways, accumulate between Days 1 and 3. Thus, their presence and type 1 pro-inflammatory profiles characterize the IM response to acute hypoxia exposure. With prolonged hypoxia exposure, we observed the emergence of a novel IM population (TLF^+^VCAM1^hi^) that upregulated *Vcam1* expression and enriched in vascular development and remodeling pathways (**Figure 3A**). By day 21 of hypoxia exposure, this population exhibited reduced expression of genes in maintaining pulmonary vascular microenvironment (*e.g.*, chemokines, *Cd163*, *Mrc1/Cd206*, *Il33*, and *Il10*) but showed activation of the classical complement pathways that can contribute to vascular dysfunction and vascular remodeling (34). Consistent with a pro-vascular remodeling functional profile, VCAM1-expressing macrophages were detected around the remodeled pulmonary arterioles after 21 days of exposure (**Figure 5**). These findings demonstrated a dynamic change in pulmonary IM transcriptional and functional profiles in hypoxia-induced PH and identified a novel population of dysregulated pro-vascular remodeling macrophages that may drive PH disease progression.

Accumulation of IMs in response to hypoxia in animal models and human PH lungs was described previously (8, 17, 20, 27). Most accumulated IMs are thought to arise from circulating monocytes (8, 20). Consistent with these observations, we did not detect a population of IMs enriched for proliferation pathways, suggesting the recruitment of circulating monocytes as the primary source of IM expansion in response to hypoxia. Both classical (CCR2^+^Ly6C^hi^) and non-classical (CX3CR1^hi^Ly6C^lo^) monocytes have been examined in the murine model of hypoxia-induced PH. Classical monocytes are reported to contribute to PH via the production of thrombospondin-1; on the other hand, specific deletion of hypoxia-inducible factor-1 in non-classical monocytes ameliorates the severity of PH (20, 21). However, how each monocyte population regulates pulmonary vascular inflammation and remodeling was unknown. Our findings suggest that these cell types may constitute different waves of cellular infiltration and have distinct roles in regulating pulmonary vascular diseases. In response to acute hypoxia exposure, the proportion of MHCII^hi^CCR2^+^EAR2^+^ increases significantly. This population expresses the highest level of *Ccr2* among the IMs and upregulates *Retnla* and *Ear2*, which have been described to be markers for recently recruited and differentiating monocytes (35), suggesting recruitment of CCR2^+^Ly6C^hi^ classical monocytes that promote acute type I inflammation in response to acute hypoxia. On the other hand, non-classical monocyte-derived macrophages have been shown to regulate vascular remodeling via chemokine secretion, classical complement activation, and TNF pathways (20). Non-classical monocytes and derived macrophages are increased in patients with altitude-induced PH and end-stage human pulmonary arterial hypertension (PAH) lung tissues (8, 36). These characteristics are consistent with TLF^+^VCAM1^hi^ populations that emerge with prolonged hypoxia exposure. While lineage tracing studies, using adoptive transfer, bone marrow chimera, or parabiotic system of CCR2/RFP (red fluorescent protein) and Cx3cr1creER/RosaGFP (green fluorescent protein) reporters with wild-type animals, are needed to establish classical and non-classical monocytes as precursors to MHCII^hi^CCR2^+^EAR2^+^ and TLF+VCAM1^hi^ directly, our findings suggest that non-classical monocytes are precursors to the TLF^+^VCAM1^hi^ pro-remodeling IMs. Thus, distinct waves of monocytes are recruited during acute and prolonged hypoxia exposure and have unique roles in regulating acute inflammation *vs.* pulmonary vascular remodeling in PH.

The early acute inflammatory response has been described in various models of PH, including our recent work in Schistosoma (20). While hypoxia induces a Type 1 inflammatory phenotype in IMs, Schistosoma induces a Type 2 inflammatory phenotype. These findings suggest divergent early IM inflammatory response types can initiate PH development. However, the acute inflammatory phase in IM largely resolves shortly after initial insults. In this study, we describe the emergence of a novel dysregulated pro-vascular modeling macrophage population with prolonged hypoxia exposure that may mediate PH disease progression after the resolution of acute inflammation in IM.

Pro-vascular remodeling macrophages have been proposed previously, but their identity and functional profiles were unknown (37). In this study, we defined a population of pro-vascular remodeling IMs by transcriptional signature and demonstrated VCAM1-expressing macrophages accumulated around the remodeled pulmonary arterioles. VCAM1 is a well-established marker for endothelial cells. However, VCAM1 expression can be upregulated in macrophages and many other cell types in inflammatory states, such as rheumatoid arthritis, cancer, transplant rejection, asthma, and atherosclerosis (38–43). Thus, future studies to isolate the TLF+VCAM1^hi^ population and define its functions will be essential to understanding how IM contributes to PH progression. Additionally, examining whether divergent early inflammatory responses lead to the emergence of a common dysregulated pro-vascular remodeling cell type will be essential in defining potential IM-targeted approaches to reverse abnormal vascular remodeling in PH. For example, the two distinct IM populations (MHCII^hi^CCR2^+^EAR2^+^ *vs.* TLF^+^VCAM1^hi^) exhibited differential activation of alternative vs. classical complement pathways (**Figure 6**, **Supplementary Figure S6**). As activation of the alternative pathway has been described in the early inflammatory phase of PH pathogenesis, it raises the possibility that MHCII^hi^CCR2^+^EAR2^+^ is a cell population that regulates the inflammatory response via its role in regulating alternative complement pathway activation (33). On the other hand, the TLF^+^VCAM1^hi^ population activates classical complement pathways and contributes to abnormal vascular remodeling. Thus, our studies suggest that distinct components of the complement system have different roles in regulating early inflammatory response and pulmonary vascular remodeling, which can be potentially targeted to modulate different phases of PH pathogenesis.

In conclusion, our study revealed dynamic shifts in IM repertoire and functional profiles in response to acute and prolonged hypoxia exposure. We identified two populations of IMs corresponding to the acute inflammatory and pro-vascular remodeling phases of the PH pathogenesis, exhibiting distinct enrichment in the inflammatory response and complement activation pathways that can be potentially targeted to modulate disease development and progression. We acknowledge limitations in this study, including those findings in the murine model system will need to be validated in human samples, the dataset is limited to IMs and does not allow analyses of how IMs may be interacting with other cells in the microenvironment, direct lineage tracing examination needed to establish distinct monocyte populations as precursors and additional animal numbers will be required to truly define pulmonary IM difference between sexes. Our findings suggest that classical and non-classical monocytes are recruited and regulate different stages of pathogenesis: early acute inflammatory responses *vs.* pulmonary vascular remodeling. We also defined a population of pro-vascular remodeling macrophages with upregulated classical complement pathways that can potentially be targeted to reverse the abnormal vascular remodeling in PH.

## Data availability statement

The datasets presented in this study can be found in online repositories. The names of the repository/repositories and accession number(s) can be found below: GSE254606 (GEO).

## Ethics statement

The animal study was approved by University of Colorado, Anschutz Medical Campus IACUC. The study was conducted in accordance with the local legislation and institutional requirements.

## Author contributions

SK: Writing – review & editing, Writing – original draft, Visualization, Validation, Software, Methodology, Investigation, Formal analysis, Data curation, Conceptualization, Project administration. CM: Writing – review & editing, Methodology, Formal analysis, Data curation, Conceptualization. RK: Writing – review & editing. RP: Writing – review & editing. NC: Writing – review & editing. HZ: Writing – review & editing. ML: Writing – review & editing. BM: Writing – review & editing, Data curation. TA: Writing – review & editing, Methodology, Data curation. BG: Writing – review & editing. Y-RY: Writing – review & editing, Writing – original draft, Visualization, Validation, Supervision, Software, Resources, Project administration, Methodology, Investigation, Funding acquisition, Formal analysis, Data curation, Conceptualization. KS: Writing – review & editing, Writing – original draft, Visualization, Validation, Supervision, Software, Resources, Project administration, Methodology, Investigation, Funding acquisition, Formal analysis, Data curation, Conceptualization.
