# Supplementary material for: Single cell transcriptomic analyses reveal diverse and dynamic changes of distinct populations of lung interstitial macrophages in hypoxia-induced pulmonary hypertension
Source: Front Immunol. 2024 Apr 15;15:1372959. doi: 10.3389/fimmu.2024.1372959 (PMC11059952; doi:10.3389/fimmu.2024.1372959)
Supplement: Supplementary file 1 [file DataSheet_1.pdf]

Supplement figure 1

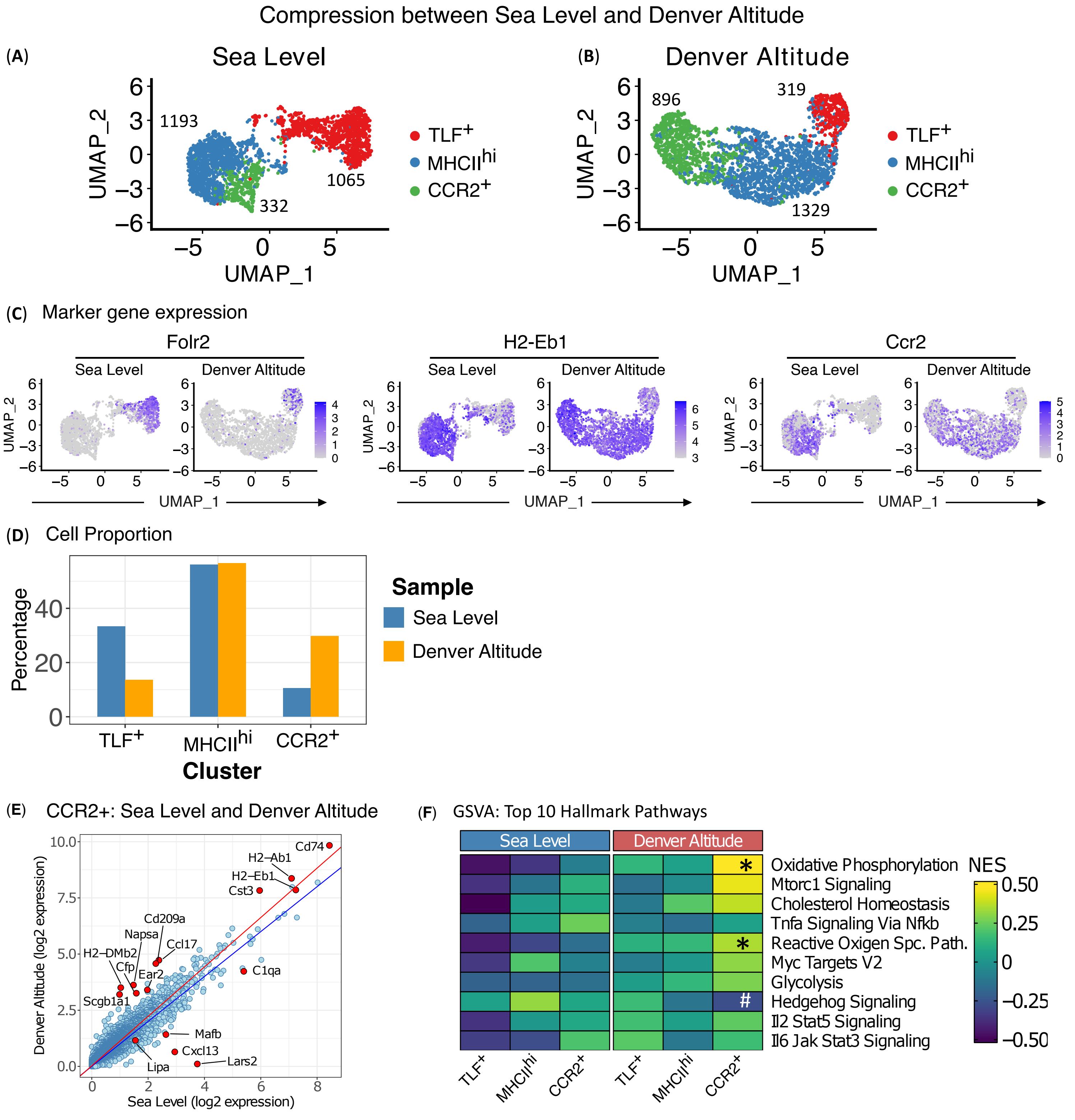

Figure S1: Comparison between IM at Sea Level and Denver Altitude. (**A and B**) Three distinct TLF⁺, MHCII^{hi}, and CCR2⁺ IM subpopulations were observed at both altitudes. (**C**) Expression of *Folr2*, *H2-Eb1*, and *Ccr2* genes was specific to clusters at both altitudes. (**D**) The proportion of CCR2⁺ cells amounted to 10% at sea level and increased to 30% at Denver altitude. The proportion of MHCII^{hi} cells remained unchanged at Denver altitude, but there was a decrease in the TLF⁺ cell proportion. (**E**) When comparing the gene profiles of the CCR2⁺ cluster between Sea Level and Denver Altitude, we observed a shift towards higher gene expression levels at Denver Altitude. The change from a slope of 1 (blue line) to 1.1 (red line) indicates an overall approximately 10% increase in gene expression at Denver Altitude. Inflammatory genes, such as Ear2, demonstrated an increase at Denver Altitude, while the anti-inflammatory gene, such as Lipa, displayed a decrease at Denver Altitude. (**F**) The Gene Set Variation Analysis (GSVA) results revealed a slightly increased Enrichment Score for inflammatory pathways (IL-2 STAT5 and IL6 Jak-STAT3) and metabolic reprogramming, including mTORC1, Glycolysis, and Oxidative Phosphorylation in CCR2⁺ cells. However, only # and * marked were statistically significant. In this context, '#' represents suppressed pathways, and '*' represents enriched pathways. IMs derived from one male and one female were used for analyses.

Supplement figure 2

(A)

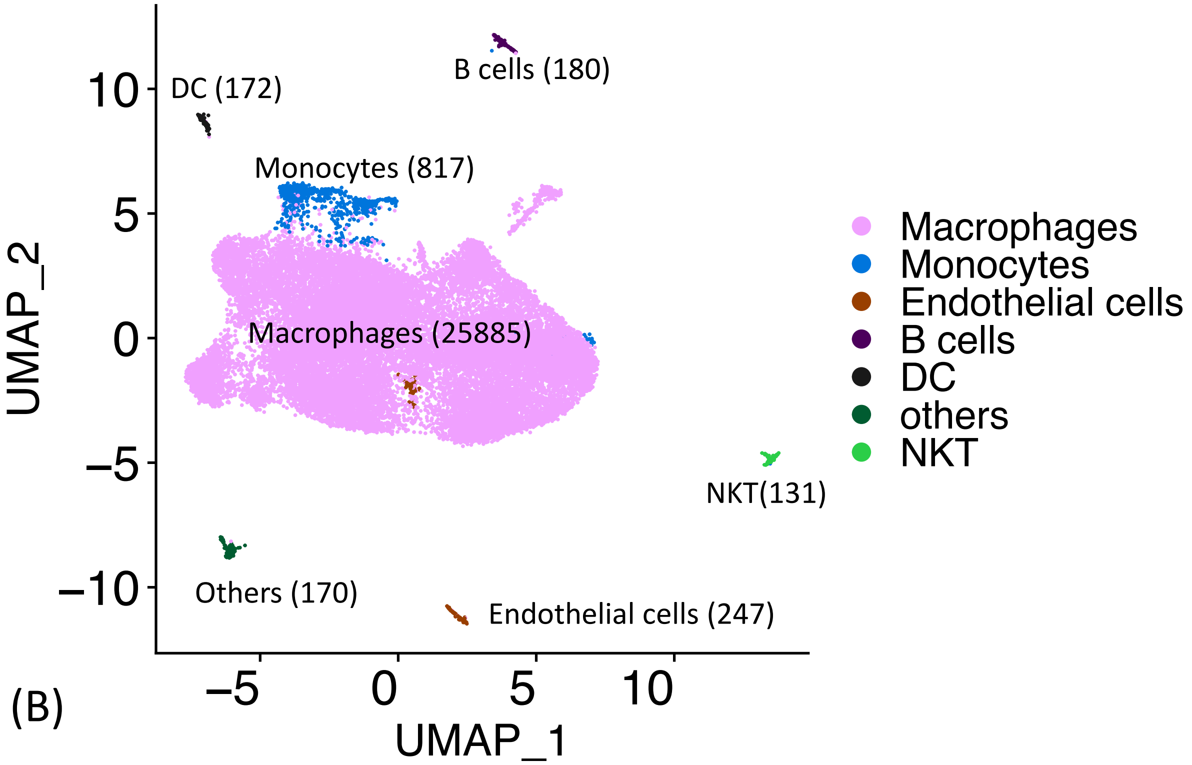

(B)

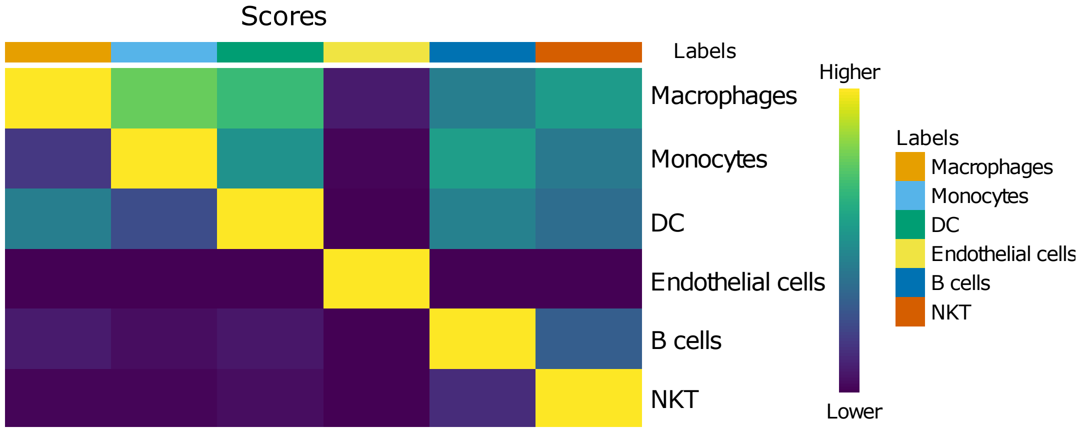

Figure S2: Utilizing the ImmGen database to categorize all clusters. (**A**) UMAP visualization showing various cell types, including macrophages, Monocytes, Endothelial cells, B cells, Dendritic cells (DC), NKT cells, and others. The number of cells for each type is indicated in parentheses. (**B**) Cluster assignment scores plot with columns representing clusters of present study and rows representing ImmGen database cells.

Supplemental Figure 3

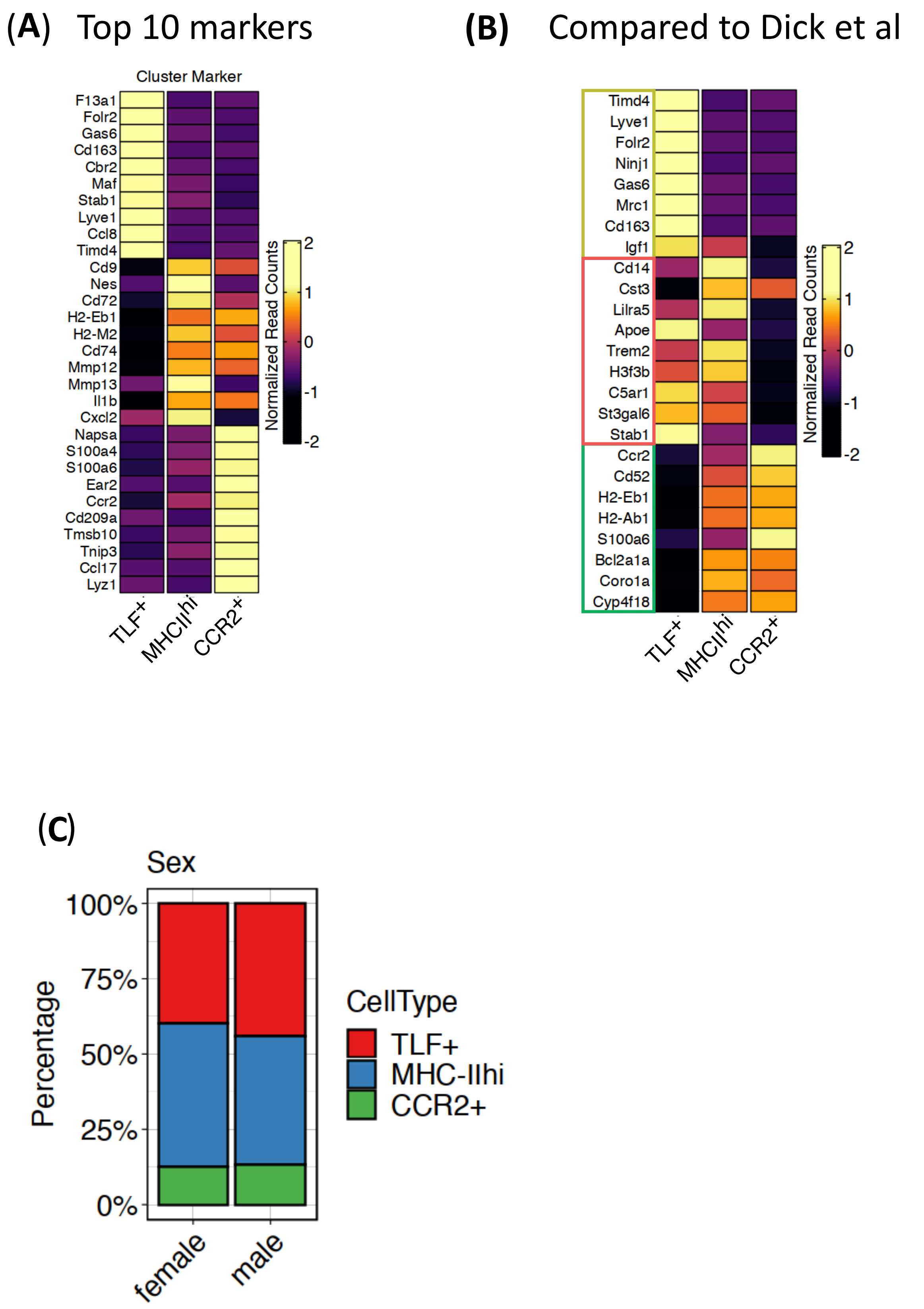

Figure S3: (**A**) Top 10 average gene expressions in TLF⁺, MHCII^{hi}, and CCR2⁺ cells. (**B**) Gene profile of the present study compared to the established method (Dick et al. 2022) for IM classification (genes ordered the same as in Dick et al. 2022). (**C**) Percentage distribution of male and female cells.

Supplement Figure 4.

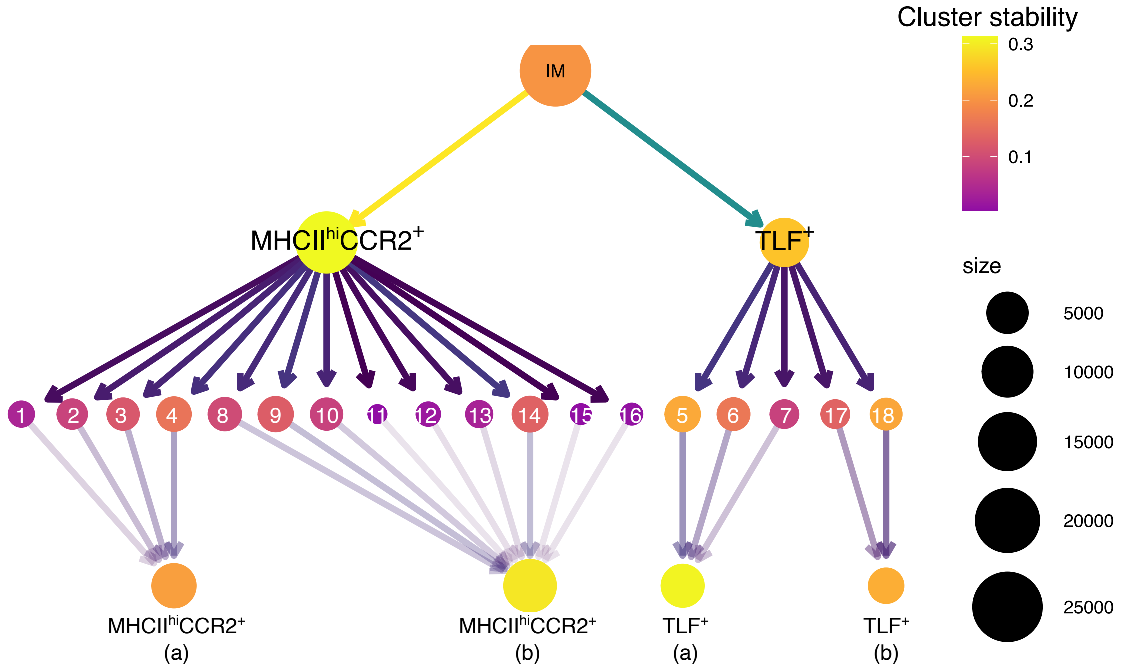

Figure S4. Cluster stability analysis provides confirmation of the presence of 4 stable clusters in response to hypoxia.

Supplement Figure 5.

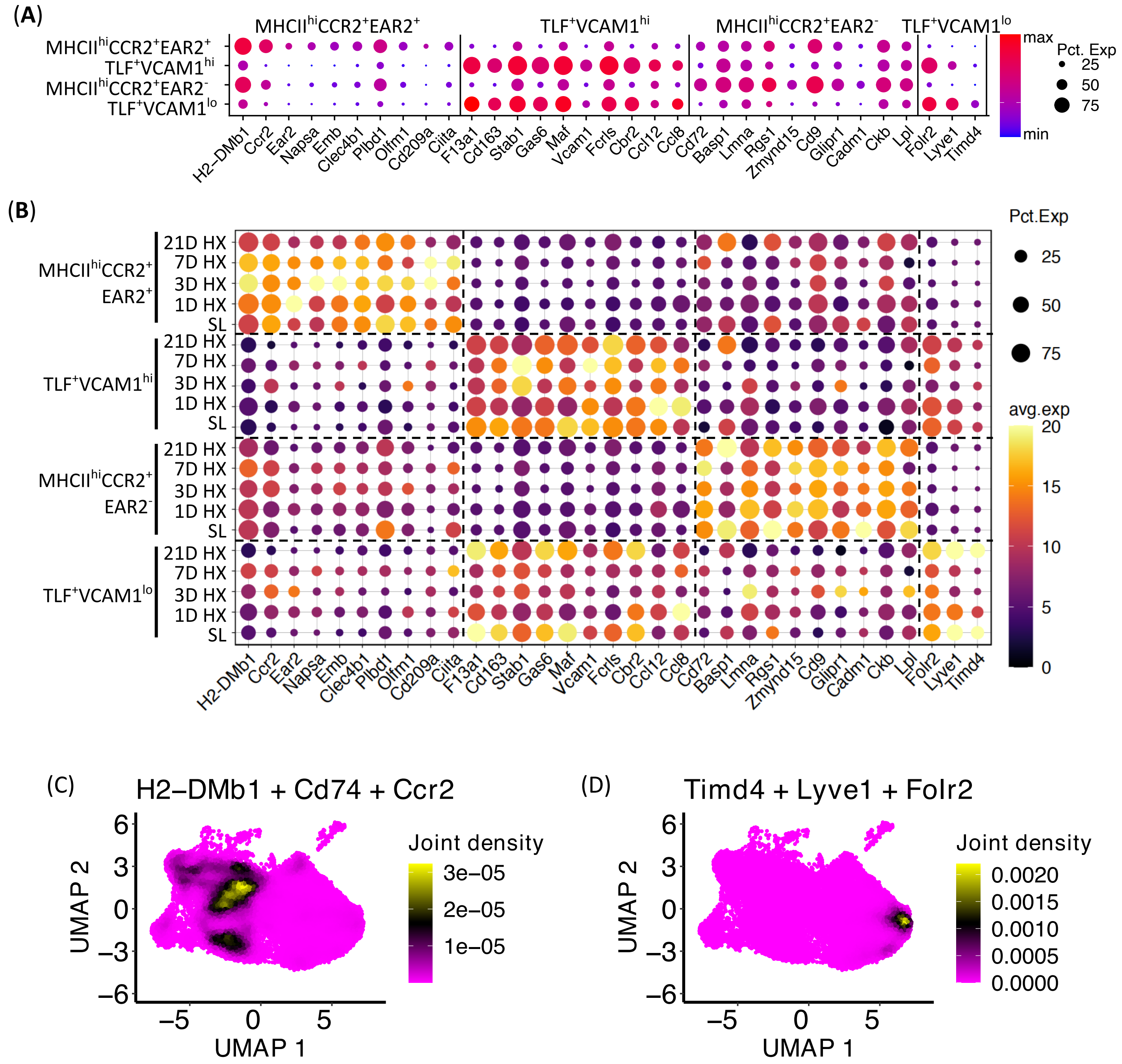

Figure S5: (**A**, **B**) Dot plot depicting the top 10 markers for the integrated IM population and at each time point. (**C**, **D**) Density plot displaying the expression of cluster-specific genes. Notably, H2-DMb1+ Cd74+ Ccr2+ genes are predominantly expressed in MHCII^{hi}CCR2^{+}EAR2^{-} cluster, while Timd4+ Lyve1+ Folr2+ genes are highly expressed in the TLF^{+}VCAM1^{lo} cluster.

Supplemental Figure 6

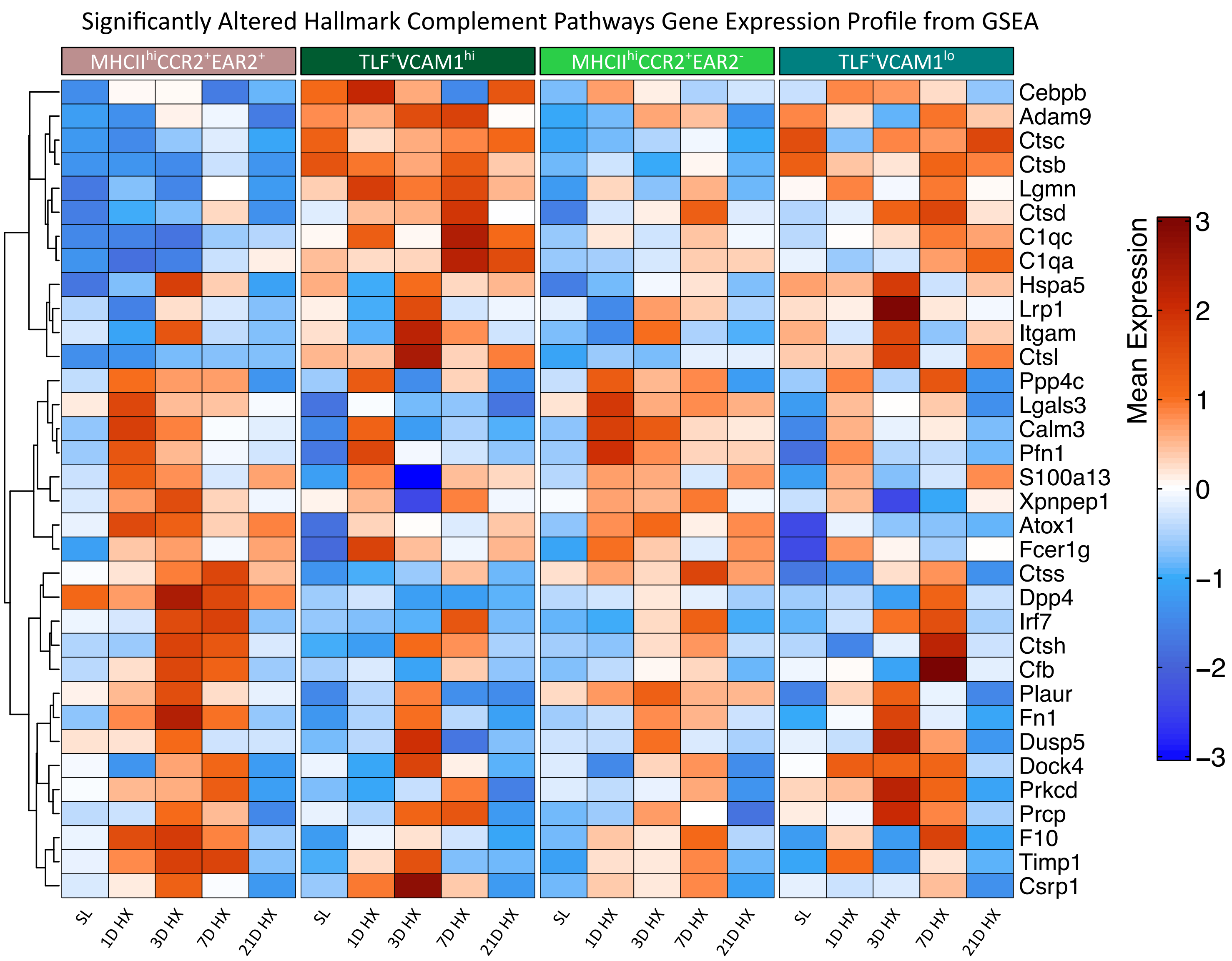

Figure S6: Distinct Gene Expression Patterns in MHCII^{hi}CCR2^{+}EAR2^{+} and TLF^{+}VCAM1^{hi}. Heatmaps of hallmark complement pathway genes, reveal distinct gene expression patterns between the MHCII^{hi}CCR2^{+}EAR2^{+} and TLF^{+}VCAM1^{hi}.
